# Draft Genome Sequence of *Mycobacterium neworleansense* Strain ATCC 49404^T^

**DOI:** 10.1128/genomeA.01314-15

**Published:** 2015-11-05

**Authors:** Shady Asmar, Catherine Robert, Olivier Croce, Aurelia Caputo, Michel Drancourt

**Affiliations:** Aix Marseille Université, URMITE, UMR 63, CNRS 7278, IRD 198, Inserm 1095, Faculté de Médecine, Marseille, France

## Abstract

*Mycobacterium neworleansense* is a rapid growing nontuberculosis species belonging to the *Mycobacterium fortuitum* complex. The draft genome of *M. neworleansense* ATCC 49404^T^ comprises 6,287,317 bp exhibiting a 66.85% G+C content, 5,997 protein-coding genes, and 89 predicted RNA genes.

## GENOME ANNOUNCEMENT

*Mycobacterium neworleansense* is a rapidly growing nontuberculous mycobacterium formerly classified as *Mycobacterium fortuitum* sorbitol-negative third biovariant (1). Within the *M. fortuitum* complex, *M. neworleansense* clusterizes with *Mycobacterium peregrinum*, *Mycobacterium septicum*, and *Mycobacterium porcicum* (2, 3). The name *M. neworleansense* derives from New Orleans, USA, where this mycobacterium was first isolated from a scalp wound (1).

We performed the whole-genome sequencing of *M. neworleansense* ATCC 49404^T^ in order to refine understanding of its taxonomic relationships with closely related species of the *M. fortuitum* complex.

From this perspective, *M. neworleansense* ATCC 49404^T^ was cultured in MGIT Middlebrook liquid culture (Becton Dickinson, Le Pont-de-Claix, France) at 37°C in a 5% CO_2_ atmosphere. *M. neworleansense* genomic DNA was then sequenced by Illumina MiSeq runs (Illumina Inc., San Diego, USA) using a 5-kb mate-paired library. Reads were trimmed using Trimmomatic (4) and assembled using Spades v3.5 (5, 6). Contigs were combined together by SSPACE v2 (7), Opera v2 (8) helped by GapFiller v1.10 (9), and homemade tools in Python to refine the set. This resulted in a draft genome consisting of nine contigs without gap for a total of 6,287,317 bp and a G+C content of 66.85%. Noncoding genes and miscellaneous features were predicted using RNAmmer (10), ARAGORN (11), Rfam (12), PFAM (13), and Infernal (14). Coding DNA sequences were predicted using Prodigal (15), and functional annotation was achieved using BLAST+ (16) and HMMER3 (17) against the UniProtKB database (18). The genome was shown to encode at least 89 predicted RNAs, including 9 rRNAs, 59 tRNAs, 1 transfer-messenger RNA (tmRNA), and 20 miscellaneous RNAs. A total of 5,997 identified genes yielded a coding capacity of 5,837,343 bp (coding percentage, 92.84%). Among these genes, 246 (4.1%) were found to be putative proteins and 1,011 (16.86%) were assigned as hypothetical proteins. Moreover, 4,296 genes matched a least one sequence in the Clusters of Orthologous Groups (19, 20) with BLASTP default parameters.

*In silico* DNA-DNA hybridation (DDH) (21) was performed with the *M. fortuitum* complex species and other reference genomes selected on the basis of their 16S rRNA gene sequence proximity with *M. neworleansense*. The *M. neworleansense* genome was locally aligned 2 by 2 using a BLAT algorithm (22, 23) against each the selected genomes, and DDH values were estimated from a generalized linear model (24). The DDH was 35.9% ± 2.48% with *Mycobacterium septicum* DSM 44393 (25), 32.8% ± 2.46% with *Mycobacterium senegalense* CK2 M4421 (1), 32.8% ± 2.46% with *Mycobacterium conceptionense* (26), 32.6% ± 2.46% with *Mycobacterium farcinogenes* (27), 32.3% ± 2.46% with *Mycobacterium peregrinum* (28), 31.7% ± 2.46% with *Mycobacterium fortuitum* ATCC 6841 (29), 20.5% ± 2.32% with *Mycobacterium chubuense* NBB4 (30) and *Mycobacterium gilvum* Spyr1 (31), and 20.1% ± 2.31% with *Mycobacterium aromaticivorans* JS19b1 (32).

These data confirm *M. neworleansense* as a unique species more closely related to *M. septicum* in the *M. fortuitum* complex.

### Nucleotide sequence accession numbers.

The *M. neworleansense* ATCC 49404^T^ genome sequence has been deposited at EMBL under the accession numbers CWKH01000001 to CWKH01000009.
